# Computerized Cognitive Training in Children With Autism and Intellectual Disabilities: Feasibility and Satisfaction Study

**DOI:** 10.2196/mental.9564

**Published:** 2018-05-25

**Authors:** Songpoom Benyakorn, Catrina A Calub, Steven J Riley, Andrea Schneider, Ana-Maria Iosif, Marjorie Solomon, David Hessl, Julie B Schweitzer

**Affiliations:** ^1^ Department of Psychiatry Faculty of Medicine Srinakharinwirot University Ongkharak, Nakhonnayok Thailand; ^2^ Department of Psychiatry and Behavioral Sciences University of California, Davis Sacramento, CA United States; ^3^ MIND Institute University of California, Davis Sacramento, CA United States; ^4^ Department of Pediatrics University of California, Davis Sacramento, CA United States; ^5^ Department of Public Health Sciences University of California, Davis Davis, CA United States

**Keywords:** autism, training, working memory, intellectual disability, treatment adherence, satisfaction

## Abstract

**Background:**

Researchers are increasingly interested in testing and developing computerized cognitive training interventions for individuals with autism spectrum disorder due to the limited accessibility of treatments for this disorder. Understanding the feasibility of testing cognitive interventions for this population is critical, especially for individuals with ASD who have low to moderate intellectual ability.

**Objective:**

The aim of the study was to evaluate the feasibility of computerized cognitive training as measured by attrition rate and a parent satisfaction survey.

**Methods:**

A total of 26 participants aged 8-17 years with an autism spectrum disorder diagnosis and significant intellectual impairment were enrolled (mean age 11.1 years). They were instructed to complete 25 sessions of Cogmed Working Memory Training in 5 to 6 weeks with coach assistance. Attrition rate and parent satisfaction surveys were measured after the completion of training.

**Results:**

Most participants (96%, 25/26) completed the training and indicated high satisfaction (>88%). However, among the participants who completed the training, 5 participants (19%) were unable to finish in 6 weeks, the recommended training period by Cogmed. Parents noted various positive (eg, voice-overs) and negative (eg, particular graphic and sounds associated with a stimulus) features of the game that they thought affected their child’s response.

**Conclusions:**

Children with autism spectrum disorder and intellectual impairments can successfully participate in computerized cognitive training interventions but may require additional weeks to complete the training beyond the time needed for children without intellectual impairments. The overall completion rate, with extended time to complete the training, was high. Developers of cognitive training programs for this population should take into account potential issues regarding the noise level of stimuli and characteristics of the visual graphics.

## Introduction

### Developing Novel Interventions for Treating Autism

Autistic spectrum disorder (ASD) is a common neurodevelopmental disorder, with an overall prevalence of 1.47% of children in the United States [1]. Traditionally, interventions for ASD have relied on models of individual training with a therapist or small group therapy with a therapist or caregiver [2]. There are inherent limitations in this model, however, due to the limited access to trained professionals and a relatively high cost of service delivery. With concerns about the rising prevalence of ASD [1], there is an urgent need for additional and supplemental interventions to address deficits associated with ASD. Increasingly, interventions using computer technology are being considered to fill this gap [3,4]. A critical step before implementing a technological intervention for persons with ASD is assessing whether its use is feasible and acceptable to caregivers. Moving new potential interventions rapidly into the community requires partnerships with the stakeholders, in which they can give feedback on their experience [5]. As noted by Kelley and colleagues [5], ongoing input from the perspective of the stakeholders (in this case, parents) is key throughout the translational phase as a potential intervention moves from inception, discovery to delivery. Input and feedback from stakeholders can then be used to develop new interventions and refine those in the pipeline. The funding agency for this project, the Department of Defense (Autism Research Program), through a Pilot Award, also recognized the need to first test the feasibility of a potential therapy before it would be tested in a full-scale, randomized control trial. Findings from feasibility studies on recruitment, retention, and satisfaction with the intervention are important to determine how and if a full-scale study should be implemented [6]. As such, this paper focuses on the feasibility and acceptability of computer technology in children with ASD who have moderate to low intellectual functioning. A future paper will present data on the efficacy of the intervention from these participants.

### Working Memory Impairments in Autism

ASD is characterized by impairments in communication skills and reciprocal social interactions, and restricted, repetitive, and stereotyped patterns of behavior and interests [7]. In addition, executive function, which is responsible for organizing and regulating behaviors [8-10], has been emphasized as a core dysfunction of ASD [10-13]. One important component of executive functioning is working memory (WM), which is involved in maintaining and manipulating incoming information during planning and executing cognitive tasks [14,15]. WM performance is highly predictive of individual differences in verbal and visuospatial memory span and reasoning [16], general fluid intelligence [17], reading comprehension difficulties [18-21], mathematical difficulties [22-24], and attention deficit hyperactivity disorder (ADHD) symptoms [25,26]. Andersen et al [27] showed that verbal WM ability in children with high-functioning ASD does not demonstrate the typical developmental increases in capacity expected over a 2-year time period [27] found in children with ADHD or typically developing (TD) children. In other studies, children with ASD had lower spatial working memory training (WMT) abilities than TD children [28,29].

Although WM capacity was previously assumed to be a stable trait [30,31], more recent studies have suggested that WM capacity can be increased through targeted training [32-35]. WMT has been shown to be beneficial in many populations, including individuals within ADHD [36-40], Down syndrome [41,42], and fetal alcohol disorders [43]. However, the efficacy of WMT is currently debated. For example, a number of reviews and meta-analyses identified limitations of the research in WMT [34,44,45], such as the lack of methodological consistency between studies [34] and inconsistency in generalization of benefits to other cognitive domains [36,44,45].

Few studies have examined the effects of computerized WMT in children with ASD [3,46]. de Vries and colleagues [3] concluded that WMT is not feasible for children with ASD, based on their sample (8-12 years of age; IQ>80) as evinced by a high attrition rate (26%) and marginal improvement on near-transfer WM and ADHD behavior. However, the findings were limited by the absence of assessment of motivation. Because high motivation and a positive attitude are critical components to successful training [32,44], it is possible that the high attrition rate may have been due to low motivation.

### Addressing Gaps in Working Memory Training in Autism

Another limitation in the existing cognitive training literature is the lack of research on children with ASD and intellectual disability (ID), which is crucial considering that over a third of individuals with ASD also have ID [1]. The aforementioned de Vries study [3] included children with intellectual functioning of greater than 80. Understanding feasibility of training is valuable for exploring the viability of translating computerized interventions, such as WMT, into clinical practice. To this end, this study assessed a computerized cognitive training program for feasibility, as measured by attrition rate and parent responses, in children with ASD with low to moderate intellectual ability.

## Methods

### Participants

**Figure 1:**
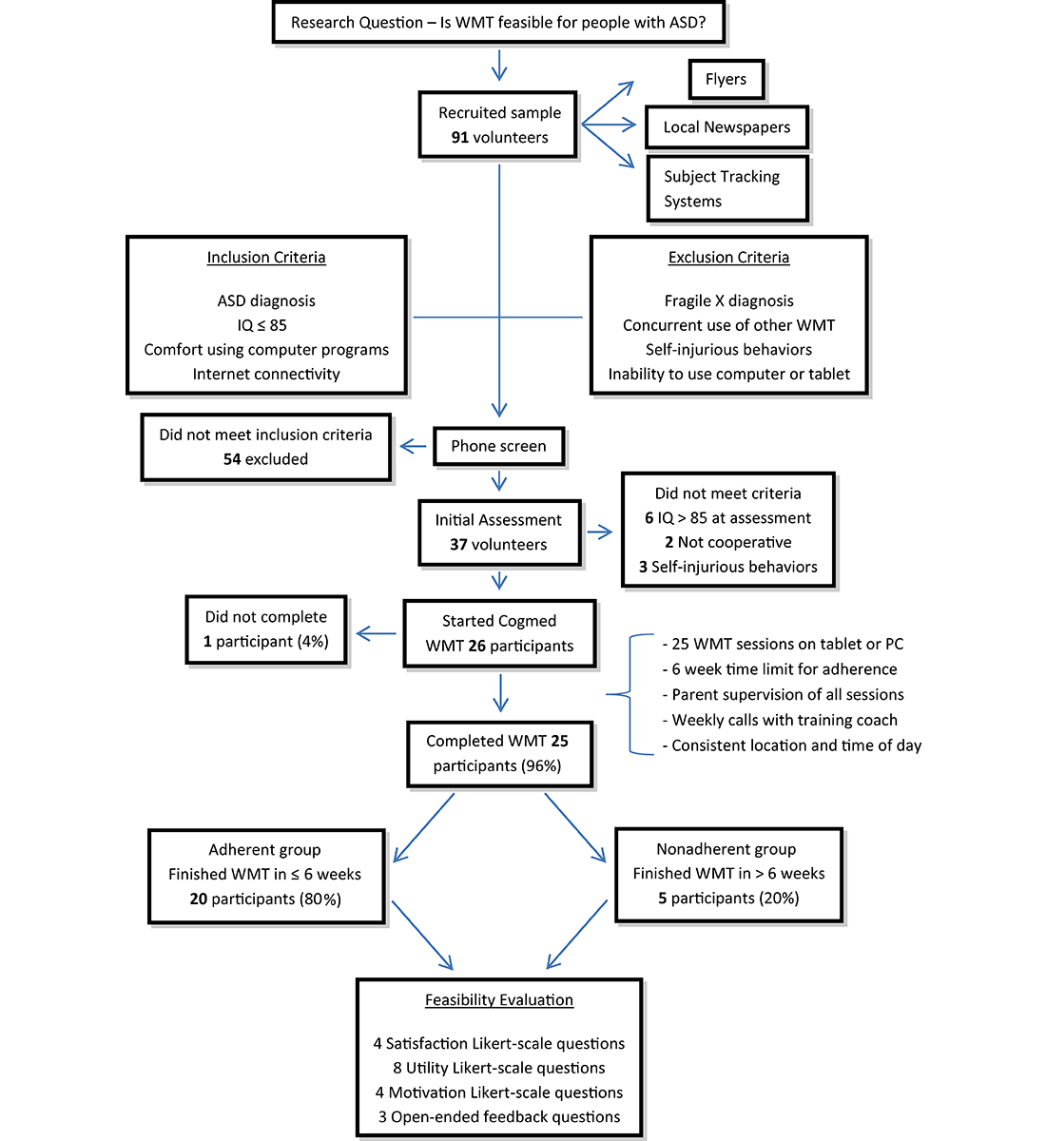
Flow of participant recruitment, participation, and outcomes assessed in feasibility study. WMT: working memory training; ASD: autistic spectrum disorder.

Participants were recruited through the MIND Institute’s Subject Tracking System, flyers located at the local clinic, Alta California Regional Center, and advertisements placed in websites and local newspapers (Figure 1). In total, 91 volunteers were assessed for eligibility via a brief series of questions with the parent of the potential participant over the phone. The inclusion criteria included children aged 8-17 years with a diagnosis of ASD, below average intellectual functioning (IQ≤85), and normal to corrected normal vision and hearing. Participants were excluded if there were plans to change current behavioral or pharmacological treatment during the course of the study, if a caregiver reported disruptive behaviors that would interfere with WMT, or if the participant was unable to use a computer or tablet. A total of 37 of the 91 volunteers met the eligibility criteria and underwent an in-person assessment visit. Participants were further excluded at the in-person assessment if observable self-injurious behaviors were present or if their IQ scores were greater than 85. Most of the volunteers (n=54) were excluded because their IQ was greater than 85. A total of 26 study participants (mean age 11.1 [SD 2.4] years) remained after exclusions. This sample included 21 males and 5 females, which reflects the ratio found in the ASD population [1]. The average IQ for the sample was 65 (range: 47-85; SD 14; Table 1).

Intellectual functioning was determined by current or previous testing, if it was available within the past 3 years with the Wechsler Intelligence Scale for Children, Fourth Edition, or Stanford-Binet Intelligence Scales, Fifth Edition (SB-5). The verbal and nonverbal routing subtests of the SB-5 were administered to estimate the abbreviated IQ for participants without recent testing. The diagnosis of ASD was confirmed by documentation of previous assessments (The Autism Diagnostic Observation Schedule or the Autism Diagnostic Interview-Revised). If no prior diagnosis had been made, ASD diagnosis was confirmed through the Social Communication Questionnaire Lifetime (SCQ), for which the total SCQ score was greater than 15.

**Table 1:** Characteristics of participants (N=26).

Variable		Summary
Age in years, mean (SD)		11.1 (2.4)
**Gender, n (%)**		
	Female	5 (19)
	Male	21 (81)
**Race^a^, n (%)**		
	Black	3 (12)
	White	14 (56)
	Asian	5 (20)
	Other	3 (12)
Intelligence quotient (IQ), mean (SD)		65 (14)
**Training device, n (%)**		
	Tablet	18 (69)
	Personal computer (PC)	8 (31)
**Cogmed version, n (%)**		
	JM^b^	15 (58)
	RM^c^	11 (42)
**Current therapies, n (%)**		
	Occupational therapy	15 (58)
	Physical therapy	4 (15)
	Speech therapy	21 (81)
	Applied behavioral analysis	11 (42)
**Psychotropic medications, n (%)**		
	ADHD^d^ stimulant	1 (4)
	ADHD nonstimulant	4 (15)
	Antidepressant	3 (12)
	Antipsychotic	6 (23)
	Mood stabilizer	3 (12)
	Other medications	2 (8)
	No medications	15 (58)
**Comorbidities, n (%)**		
	ADHD	6 (23)
	Mood disorders	2 (8)
	Anxiety disorders	3 (12)
	Seizure disorders	3 (12)
	Other mental disorders	2 (8)
	Other physical disorders	2 (8)
**Completion and adherence, n (%)**		
	Completed training^e^	25 (96)
	Adherent to protocol^f^	19 (73)
	Nonadherent^f^	5 (19)
	Did not complete training^e^	1 (4)
	Combined attrition^g^	6 (23)

^a^n=1 did not provide an answer.

^b^JM: Cogmed for preschool-aged children.

^c^RM: Cogmed for school-aged children.

^d^ADHD: attention deficit hyperactivity disorder.

^e^Required completing 25 sessions as reported by the Cogmed website.

^f^Required completion of 25 sessions within 6 weeks of start date.

^g^Not completing training or not adhering to protocol.

### Procedure

All procedures of the study were reviewed and approved by the University of California, Davis Institutional Review Board. After screening for criteria with the parents over the phone, parents provided written informed consent and participants over the age of 11 years provided assent. Tests assessing baseline WM, cognitive abilities, attentional functioning, and autism symptoms were administered at the participants’ homes. After participants completed Cogmed training, researchers administered the same test battery to assess training effects. Data on training effects on objects measures of performance and rating scales will be presented in a separate publication. Parents completed posttraining surveys to evaluate feasibility of WMT. Participants were reimbursed US $20 for their time in the initial assessment and US $50 for the follow-up assessment.

### Intervention

All participants were instructed to complete 5 Web-based Cogmed Working Memory Training [47] sessions per week for 5 weeks, for a total of 25 training sessions. There were 2 difficulty levels for participants. Cogmed RM was developed for school-aged TD children, whereas Cogmed JM was for preschool TD children. Those who comprehended 9 of the 11 Cogmed RM games, as determined by researchers during the in-person assessment, were assigned to Cogmed RM (n=15), with the remainders assigned to Cogmed JM (n=11). The Cogmed JM groups required training for 15 min per session and the Cogmed RM group required training for 30 min per session. Participants were trained either on the Cogmed tablet app (n=18; use of finger for item responses), with tablets provided as necessary, or on the Cogmed website (n=8; use of PC with a mouse for item responses). See Table 1 for additional information.

Each training session was conducted at home in a location with limited distractions and parental supervision. As per the Cogmed’s protocol, parents were trained as training aides by Cogmed coaches to ensure participants’ motivation and focus. Researchers who underwent Cogmed Research Coach Training contacted parents by phone or email at least once a week to address concerns related to training. These coaches had online access to participants’ frequency of use and performance on Cogmed tasks to track progress and provide feedback as necessary. Coaches also answered questions regarding software issues and took general feedback about use of the program.

Both Cogmed versions involved visuospatial WM span tasks, and, additionally, the RM version included verbal WM span tasks. Daily Cogmed JM training involved completing 3 of the 7 JM games, whereas the RM training involved the completion of 8 of the 10 RM games, with games rotated in each session to maintain novelty and interest. Both versions were adaptive; the difficulty gradually increased after correct trials and decreased after incorrect trials. Both versions emitted auditory and visual feedback after each trial to indicate success or failure at the task. After the completion of each training session, Cogmed JM users received a virtual fish for their digital aquarium, and Cogmed RM users played a racing game as a reward. For added motivation, parents gave a sticker after each session for a reward chart, and parents and participants decided on daily, weekly, and full training completion rewards.

### Measures

#### Completion, Adherence, and Combined Attrition

The completion rate was defined as the percentage of participants who completed 25 sessions of Cogmed WMT. Although it is recommended by Cogmed that training should be completed in 5 weeks, training duration was extended to 6 weeks; it was presumed that participants would require more time to complete the training due to learning and behavioral challenges associated with ASD and ID. Participants were considered “adherent” if they finished 25 sessions within 6 weeks. Participants who took longer than 6 weeks to complete the training were considered “nonadherent” with the training protocol. The combined attrition rate was defined as the percentage of participants who did not complete 25 sessions and who were nonadherent with the training protocol.

#### Posttraining Survey

At the end of training, parents completed a questionnaire with 16 Likert-scale and 3 open-ended questions. The questionnaire, adapted from a previous ADHD intervention study [48], aimed to measure subjective opinions regarding the feasibility of the program. Likert-scale questions comprised a 5-point scale (1=strongly disagree, 2=disagree, 3=neutral, 4=agree, and 5=strongly agree) and inquired into participant motivation, parent satisfaction, and perceived utility. We summarized the responses by calculating both mean and SD and the percentage of parents who answered agree/strongly agree (satisfied/strongly satisfied), were neutral, or answered disagree/strongly disagree (dissatisfied/ strongly dissatisfied) for each question. Open-ended questions were asked about the pros and cons of the program and possible improvements.

### Statistical Analysis

Data were collected and managed using REDCap electronic data capture tools hosted at University of California, Davis [49]. Feasibility of Cogmed training was assessed both quantitatively and qualitatively. Quantitatively, descriptive statistics were calculated for the completion rate and Likert-scale questions. Qualitatively, deidentified data from open-ended questions were used for content analysis. Initially, phrases taken from the open-ended responses were tagged with low-level codes. These codes were compared and found to fit into 3 broad categories based on previous qualitative literature in computer-based training [50]: technological features, personal experiences, and strategy of use.

## Results

### Attrition

A total of 25 of the 26 participants (96%) completed all 25 sessions of Cogmed WMT. Of these 26 participants, 20 (77%) completed the WMT within 6 weeks and were considered adherent to protocol, 5 (19%) took more than 6 weeks and were considered nonadherent, and 1 participant (4%) completed only 16 sessions due to unsolved technical problems. The combined attrition rate (counting those who did not complete 25 sessions and did not complete the training within 6 weeks) was 23%. The combined attrition rates were similar across Cogmed JM and RM users. Of the 15 JM participants, 3 were considered nonadherent and 1 did not complete the training (combined attrition rate=27%). All 11 RM participants completed the training, but 3 were considered nonadherent (combined attrition rate=27%).

### Posttraining Ratings

Likert-scale questions queried parents about the 3 domains of satisfaction, perceived utility, and motivation (Table 2). The percentage of parents who agreed or strongly agreed with questions regarding program satisfaction was at least 88% for each item. Responses to questions about the perceived utility of the training were less consistent. There was mixed evidence about improvements in daily life (child’s attention/behavior, peer relations, study skills, homework, self-esteem/attitude, and relationship with the parent), but most parents reported that they observed improvement on performance on the program itself (76% agree) and felt the WMT approach was appropriate for their child (84% agree). Regarding motivation, parents generally thought that their children found Cogmed to be enjoyable, with 72% of respondents agree to this item. However, many parents found it hard to maintain their children’s motivation to train on a daily basis. The item asking whether it was easy to keep the child motivated, received a high number of “disagree” responses (28%); similarly, only 32% of parents said that the child’s motivation improved by end of training.

The pattern of parent responses was similar for Cogmed JM and RM versions, with a few exceptions. Parents of children assigned the JM version perceived more improvement in motivation and less improvement in homework (Multimedia Appendix 1). It is important to note, however, that only 19 of the 25 parents responded on the item asking about child's homework improvement, as some children were not assigned homework during the summer months of data collection. We also examined (data not shown) the responses of the 5 participants with comorbid ADHD who completed the training, and they were consistent with the pattern observed for those without comorbid ADHD.

### Posttraining Feedback

Content in open-ended responses was analyzed and classified into 3 main areas: (1) technological features, (2) personal experiences, and (3) usage strategies.

#### Technological Features

Many of the parents had comments about game design, system functionality, and rewards. Comments about game features included discussion about the graphics and sound. One parent said her child enjoyed the “giggling sounds”; multiple participants liked the voice-over. Moreover, parents liked the reward games that children accessed when all daily tasks were finished. However, participants also reacted negatively to some stimuli, such as the cow sounds or spider graphics. Another area that parents noted as need improvement was game variability. Several parents indicated that motivation could be improved either by varying the games or by giving the child a choice about which game to play each day. Moreover, another common theme among technology-related comments was that there were “glitches” or “bugs” in the Cogmed WMT website.

#### Personal Experiences

Several parents indicated that the WMT affected daily functioning in their children. Several described perceived increased abilities in their children associated with using WMT, such as increased focus, memory, and reaction time. However, some parents saw strong negative emotional reactions in their children. Tantrums, low motivation, and frustration were all reported, though pride at completing difficult tasks was also mentioned.

#### Usage Strategies

Cogmed has several strategies to help improve adherence and outcomes, as described in the Methods section; these strategies generated many comments. For example, imposing breaks could be difficult or turn into a power struggle. Several comments mentioned appreciation of the coaches. Responses to the daily training length varied, with some parents wishing for shorter sessions and others wanting longer.

**Table 2:** Summary of parent posttraining ratings for 25 participants who completed all 25 sessions of Cogmed working memory training.

Item^a,b^		Mean (SD)	Frequency (%)^c^		
			Agree^d^	Neutral^e^	Disagree
**Satisfaction**					
	Staff show interest and concern	4.52 (0.59)	24 (96)	1 (4)	0 (0)
	Staff are skilled	4.56 (0.58)	24 (96)	1 (4)	0 (0)
	Treatment is of high quality	4.24 (0.93)	22 (88)	2 (8)	1 (4)
	I would recommend this to others	4.20 (0.58)	23 (92)	2 (8)	0 (0)
**Perceived utility**					
	Child's attention/behavior improved	3.56 (0.65)	14 (56)	10 (40)	1 (4)
	Child's peer relations improved	3.32 (0.63)	8 (32)	16 (64)	1 (4)
	Child's study skills improved^f^	3.35 (0.57)	7 (30)	16 (70)	0 (0)
	Child's homework improved^g^	3.47 (0.61)	8 (42)	11 (58)	0 (0)
	Child's self-esteem/attitude improved	3.32 (0.63)	8 (32)	16 (64)	1 (4)
	Relationship with child improved	3.44 (0.58)	10 (40)	15 (60)	0 (0)
	Child made progress in training	3.88 (0.60)	19 (76)	6 (24)	0 (0)
	WMT^h^ approach is appropriate	3.96 (0.68)	21 (84)	3 (12)	1 (4)
**Motivation**					
	Child enjoyed the training	3.76 (0.78)	18 (72)	5 (20)	2 (8)
	Easy to keep child motivated	3.44 (1.08)	14 (56)	4 (16)	7 (28)
	Training is as enjoyable as commercial games	2.64 (0.95)	4 (16)	6 (24)	15 (60)
	Child’s motivation improved by end of training	2.88 (1.01)	8 (32)	6 (24)	11 (44)

^a^Ratings scale: 1=strongly disagree, 2=disagree, 3=neutral, 4=agree, 5= strongly agree.

^b^Item descriptions paraphrase actual questions.

^c^Percentages may not add to 100% due to rounding.

^d^Percent agree indicates percentage of sample responding with 4 or 5.

^e^Percent disagree indicates percentage of sample responding with 1 or 2.

^f^n=2 answered not applicable.

^g^n=6 answered not applicable.

^h^WMT: working memory training.

## Discussion

The purpose of this pilot study was to examine, for the first time, the feasibility of using a cognitive training program in children with ASD with low to moderate intellectual functioning. Although the vast majority of participants (96%) were able to complete the 25 Cogmed sessions, many of them (24%) needed more than 6 weeks, which is an extension of the 5-week period recommended by Cogmed. This suggests that although completion may be feasible for children with ASD, Cogmed’s suggested training schedule may be too aggressive for this population.

Using a strict time requirement of completion within 6 weeks resulted in an attrition rate similar to the attrition rates found in previous research on children with ASD and no intellectual impairment (26%) [3] and children with ADHD (23%) [36]. In these previous studies, it is unclear whether participants were given the option of finishing training beyond 6 weeks; thus, the overall completion rate in this study (96%) is not directly comparable. However, WMT studies that incorporated a flexible time requirement with other clinical populations reported similar completion rates. For example, Conklin et al [51] found that 88% of childhood cancer survivors completed training in 5-9 weeks. Also of note, Mawjee et al [52] found that children with ADHD had a higher Cogmed completion rate (78%) and showed more motivation when given shorter training lengths (15 min/session) than did those who were assigned standard-length training (45 min/session; 44% completion). In this study, participants assigned the JM version (15 min/session) had similar completion rates as those assigned the RM version (30 min/session), but similar to the findings of Mawjee et al [52], parents of children assigned the JM version perceived more improvement in their child’s motivation than those assigned the RM version.

The sample in this study had significant levels of cognitive delay (mean IQ=65), and thus may have required more time to complete the program due to the presence of learning difficulties. In support of this idea, Bennett et al [41] showed that a sample of children with Down syndrome who had a mean IQ less than 70 took 10-16 weeks to complete Cogmed WMT. Therefore, an appropriate training schedule should be further investigated for successful WMT implementation in populations with developmental delays.

Qualitative measurement of feasibility, as measured by parental survey, revealed overall satisfaction with the WMT. More than 85% of parents agreed with each satisfaction-related item, which is similar to findings from other studies that evaluated the feasibility of WMT in other populations [51,53]. On the basis of the open-ended responses in this study, it appears that in-home training, flexibility to set a preferred training time, and weekly coaching calls contributed to higher parent satisfaction. Although most parents reported that their children enjoyed the training, only half felt it was easy to keep their children motivated. This may be due to training difficulty, length of training (eg, RM vs JM), or technical problems.

Parents consistently reported agreement with items regarding perceived improvement in child attention and behavior and progress in training, but they reported mainly neutral responses to questions regarding improvement in academic or interpersonal relationship skills. This finding is consistent with the results in the study by Chacko et al [36], which showed Cogmed resulted in improvement in WM but did not transfer to improvement in daily life skills.

This study cannot make any conclusions regarding the effectiveness of WMT in children with ASD and ID. The goal of this study was to determine whether this population can engage in cognitive training in a home setting to permit future evaluation of the effectiveness of training. A future manuscript will present findings on the effectiveness of this training.

On the basis of the findings from the parent surveys, the authors suggest the following solutions for the successful implementation of computerized cognitive training programs for children with ASD. First, the training program should be deployable in such a way as to be nonaversive to children who have sensitivities to visual and auditory stimuli. Due to sensory processing issues, which are often found in children with ASD, some parents reported that their children found certain graphics (eg, spiders) and sounds (eg, “Boo” after an incorrect response) distressing. These stimuli were unavoidable; games could not be skipped and participants were not able to turn off sounds due to program’s use of auditory instructions. WMT programs should give users the option to edit graphics and sound settings or to skip games that induce distress.

Second, technical problems should receive high priority. Due to the characteristics associated with ASD, such as inflexibility and difficulty with routine change [7], technical problems may interfere more with adherence and motivation among individuals with ASD than among other populations. Indeed, open-ended responses showed that these problems often triggered frustration. The only participant who did not complete the training program experienced a software crash, which erased the record of his work, causing him to quit permanently. Individuals with ASD may require a training program that is more fault-tolerant and less liable to crash.

Third, the use of behavior management techniques may facilitate adherence with computer training in this population. Certain behavioral problems such as temper tantrums and self-injurious behavior can hinder training. Although the coaches provided guidance on reinforcement techniques, additional behavioral strategies such as stimulus control, extinction, or relaxation training may further decrease the likelihood of disruptive behaviors and increase adherence to protocol.

Fourth, the training should be more intrinsically motivating to maintain the interest of children with ASD and ID. Because this study utilized the adaptive version of Cogmed, in which the difficulty level gradually increased after correct consecutive trials, participants were working at peak level at all times. To keep the interest, it is crucial that the training is also highly motivating. The posttraining surveys indicate that keeping participants motivated to use Cogmed WMT was difficult, and that Cogmed was not as enjoyable as commercially available games. We suggest that both of these issues can be improved with better game design; program developers should create more motivating gameplay or use interesting characters, stories, and goals to maintain participants focus.

### Limitations

Although this study offers novel information on a population that has been understudied in the cognitive training research, the findings must be interpreted in light of limitations. Because there was no control group, self-report biases may have inflated some feasibility responses. Future studies should consider a systematized qualitative design, such as a focus group, to provide more comprehensive information. Moreover, the sample size was relatively small; thus, increasing the number of participants in future research may further enhance the generalizability of the present findings. Future research should also consider including more severe cases of ASD, as some individuals were excluded due to self-injurious behavior or high levels of noncompliance.

The funding for this project was specifically given by the agency to fund feasibility projects to assess if potential interventions would be successful and worth exploring in larger, controlled design for individuals with autism. This paper met those requirements in addition to a sister manuscript, which is under preparation, assessing the effects of the cognitive training on objective measures of performance and rating scales.

### Conclusions

Assessing stakeholder perspectives in intervention research is critical throughout the design and implementation process. The development of computerized cognitive training procedures for children with ASD is likely to increase due to their accessibility. This feasibility study is the first to systematically evaluate the feasibility and parent evaluation of cognitive training among children with ASD and ID. Findings suggest greater feasibility if a flexible training schedule is used in future efficacy studies. In addition, although most parents reported satisfaction with the program assessed in this project, many parents reported software glitches and poor tolerance of graphics and sounds by their children. This may be an issue more unique to children with ASD. Thus, parent satisfaction may be improved if users are given greater control over the graphics, sounds, and interactions with specific stimuli. Developers of gaming software for training in ASD would be wise to employ focus groups to gather feedback on how the game stimuli are perceived. Future research will need to assess the efficacy, effectiveness, and generalizability of computerized cognitive interventions for children with ASD.

## Appendix

Multimedia Appendix 1 Summary of parent posttraining ratings by Cogmed version.

## Abbreviations

ADHD: attention deficit hyperactivity disorder
ASD: autistic spectrum disorder
ID: intellectual disability
JM: Cogmed for preschool-aged children
RM: Cogmed for school-aged children
SB-5: Stanford-Binet Intelligence Scales, Fifth Edition
SCQ: Social Communication Questionnaire
TD: typical developing
WM: working memory
WMT: working memory training
