# Supplementary material for: Computerized Cognitive Training in Children With Autism and Intellectual Disabilities: Feasibility and Satisfaction Study
Source: JMIR Ment Health. 2018 May 25;5(2):e40. doi: 10.2196/mental.9564 (PMC5993974; doi:10.2196/mental.9564)
Supplement: Multimedia Appendix 1 [file mental_v5i2e40_app1.pdf]

Summary of parent posttraining ratings for 25 participants who completed all 25 sessions of Cogmed working memory training by Cogmed version.

Item[a,b]	Cogmed Version							
	JM[c] (n=14)				RM[d] (n=11)			
	Mean (SD)	Percentage[e]			Mean (SD)	Percentage[e]		
		Agree[f]	Neutral	Disagree[g]		Agree[f]	Neutral	Disagree[g]
**Satisfaction**								
1. Staff show interest and concern	4.36 (0.63)	93	7	0	4.73 (0.47)	100	0	0
2. Staff are skilled	4.43 (0.65)	93	7	0	4.73 (0.47)	100	0	0
3. Treatment is of high quality	4.07 (1.14)	79	14	7	4.45 (0.52)	100	0	0
4. I would recommend this to others	4.21 (0.58)	93	7	0	4.18 (0.60)	91	9	0
**Perceived utility**								
5. Child's attention/behavior improved	3.50 (0.52)	50	50	0	3.64 (0.81)	64	27	9
6. Child's peer relations improved	3.29 (0.47)	29	71	0	3.36 (0.81)	36	55	9
7. Child's study skills improved[h]	3.29 (0.47)	29	71	0	3.44 (0.73)	33	67	0
8. Child's homework improved[i]	3.25 (0.45)	25	75	0	3.86 (0.69)	71	29	0
9. Child's self-esteem/attitude improved	3.21 (0.43)	21	79	0	3.45 (0.82)	45	45	9
10. Relationship with child improved	3.36 (0.50)	36	64	0	3.55 (0.69)	45	55	0
11. Child made progress in training	3.64 (0.50)	64	36	0	4.18 (0.60)	91	9	0
12. WMT approach is appropriate	4.14 (0.53)	93	7	0	3.73 (0.79)	73	18	9
**Motivation**								
13. Child enjoyed the training	3.64 (0.93)	64	21	14	3.91 (0.54)	82	18	0
14. Easy to keep child motivated	3.50 (0.94)	64	14	21	3.36 (1.29)	45	18	36
15. Training is as enjoyable as commercial games	2.64 (1.01)	21	14	64	2.64 (0.92)	9	36	55
16. Child’s motivation improved by end of training	3.14 (0.86)	43	29	29	2.55 (1.13)	18	18	64

[a]Ratings scale: 1=strongly disagree, 2=disagree, 3=neutral, 4=agree, 5= strongly agree.
[b]Item descriptions paraphrase actual questions.
[c]JM: Cogmed for preschool-aged children.

[d]RM: Cogmed for school-aged children.
[e]Percentages may not add to 100% due to rounding.
[f]Percent agree indicates percentage of sample responding with 4 or 5.
[g]Percent disagree indicates percentage of sample responding with 1 or 2.
[h]n=2 answered not applicable.
[i]n=6 answered not applicable.
